# CaNAC61, CaNAC79, and CaNAC92 Act as Negative Regulators in Pepper Defense Response Against *Phytophthora capsici*

**DOI:** 10.3390/biology15120943

**Published:** 2026-06-17

**Authors:** Yu Wang, Moli Chu, Beibei Gong, Xueqi Li, Jie Wang, Muhammad Azeem, Yawei Li, Wei Cheng

**Affiliations:** 1 College of Life Sciences, Anhui Normal University, Wuhu 241000, China; 2 Anhui Provincial Key Laboratory of Molecular Enzymology and Mechanism of Major Metabolic Diseases, Anhui Normal University, Wuhu 241002, China; 3 Anhui Provincial Key Laboratory of the Conservation and Exploitation of Biological Resources, Anhui Normal University, Wuhu 241002, China; 4 Anhui Provincial Engineering Research Centre for Molecular Detection and Diagnostics, Anhui Normal University, Wuhu 241002, China

**Keywords:** *Capsicum annuum*, *Phytophthora capsici*, NAC transcription factor, disease resistance, negative regulator

## Abstract

Phytophthora blight, caused by the oomycete pathogen *Phytophthora capsici*, is a devastating disease that severely constrains pepper (*Capsicum annuum*) production, leading to significant yield reduction and quality deterioration. Pathogen infection elicits a host immune response that involves extensive transcriptional reprogramming, during which transcription factors (TFs) act as key regulatory hubs linking upstream signaling cascades to downstream defense gene expression networks. NAC TFs represent a plant-specific gene family and play crucial roles in plant growth, development, and response to various stresses. However, the infection-responsive transcriptional dynamics and functions of NAC TFs during pepper–*P. capsici* interactions remain poorly elucidated. In this study, transcriptome profiling and RT-qPCR analysis of pepper plants challenged with *P. capsici* identified three NAC TF genes—*CaNAC61*, *CaNAC79*, and *CaNAC92*—that were consistently upregulated at the infection stages. Subcellular localization assays demonstrated that all these three proteins localize to the nucleus. Silencing of *CaNAC61*, *CaNAC79*, or *CaNAC92* in pepper conferred enhanced resistance to *P. capsici*. In contrast, their transient overexpression in pepper leaves significantly promoted lesion expansion and suppressed transcript levels of the defense marker genes *CaPR1*, *CaDEF1*, and *CaLOX1*. Consistently, heterologous overexpression in transgenic *Nicotiana benthamiana* further validated *CaNAC61*, *CaNAC79*, and *CaNAC92* as negative regulators in resistance to *P. capsici*. Collectively, our findings demonstrated that CaNAC61, CaNAC79, and CaNAC92 negatively regulate plant resistance to *P. capsici*, expanding the functional diversity of NAC TFs in plant immune responses and providing valuable candidate targets for genetic improvement against Phytophthora blight.

## Simple Summary

Phytophthora blight of pepper is a major disease that severely reduces crop yield and quality. NAC transcription factors are known to play key roles in plant growth, development, and stress responses, but their contributions to pepper resistance against *Phytophthora capsici* remain largely unknown. In this study, three NAC transcription factors—CaNAC61, CaNAC79, and CaNAC92—were identified as strongly induced during *P. capsici* infection and were shown to be localized in the nucleus. Gain- and loss-of-function analyses demonstrated that these genes act as negative regulators of pepper resistance against *P. capsici*. These findings provide new insights into the molecular mechanisms underlying pepper defense and highlight potential targets for the development of disease-resistant pepper cultivars.

## 1. Introduction

Pepper is an economically important vegetable crop valued for its culinary, coloring, and medicinal uses [1,2]. Phytophthora blight, caused by the oomycete pathogen *P. capsici*, is one of the most destructive diseases affecting pepper production [3,4]. Under warm and humid environmental conditions, *P. capsici* spreads rapidly and can infect multiple plant organs, including roots, stems, leaves, and fruits, leading to substantial yield losses and even plant mortality [5,6]. Current management strategies for this disease rely heavily on cultural practices (e.g., crop rotation) and chemical control; however, field efficacy is often inconsistent and inadequate under conditions highly favorable for pathogen infection [3]. Breeding for disease resistance is regarded as a more economical, effective, and sustainable strategy; however, germplasm resources for pepper resistance against *P. capsici* are limited, and the resistance is often quantitative with a complex genetic basis. These characteristics complicate the genetic improvement process and restrict the availability of stable, highly resistant commercial cultivars [3,4]. Therefore, there is an urgent need to identify valuable candidate genes in pepper’s defense response to *P. capsici*.

Plants have evolved a multilayered immune system through long-term coevolution with pathogens, which is broadly divided into pattern-triggered immunity (PTI) and effector-triggered immunity (ETI) [7]. Accumulating evidence indicates that PTI and ETI are not isolated pathways but function in a coordinated and integrated manner to activate downstream defense responses and restrict pathogen growth [8]. At the molecular level, immune signaling ultimately drives large-scale transcriptional reprogramming, which induces or represses numerous defense-related genes to initiate and fine-tune plant immune responses [9,10]. In this process, transcription factors (TFs) act as central regulatory hubs connecting upstream immune signaling cascades to downstream gene expression networks by recognizing cis-elements within target gene promoters and regulating their transcription, thereby mediating robust initiation, amplification, and fine-tuning of plant immune responses [10,11]. Multiple plant TF families—including AP2/ERF, MYB, bZIP, WRKY, bHLH, and NAC—have been identified as critical regulators of defense-related transcriptional reprogramming, collectively modulating plant defense responses against pathogen infection [12,13].

NAC (NAM/ATAF/CUC) transcription factors constitute a large plant-specific transcription factor family, typically characterized by a conserved N-terminal NAC domain and a divergent C-terminal transcriptional regulatory region [14]. NAC TFs not only participate in organ development, senescence, and adaptation to abiotic stress [15,16] but also function extensively in regulating plant immune responses against pathogen infection [12,17]. During plant–pathogen interactions, NAC TFs often act as important nodes in immune regulatory networks and modulate plant disease resistance via diverse modes of action [12,18]. For example, in tomato, SlNAP1 directly activates downstream genes involved in hormone metabolism, thereby enhancing resistance to *Pseudomonas syringae pv*. *tomato* (Pst) and *Ralstonia solanacearum* [12,19]. In diploid wheat, TuNAC69 positively regulates NLR (nucleotide-binding leucine-rich repeat)–mediated stripe rust resistance [20]. In addition, the RxLR effector Pi03192 from *Phytophthora infestans* targets the potato NAC TFs NTP1/NTP2 and promotes pathogen infection by inhibiting their translocation from the endoplasmic reticulum to the nucleus [21]. Conversely, some NAC TFs function as negative immune regulators: *Arabidopsis* ATAF1 (ANAC002) has been validated to negatively regulate defense responses against bacterial and necrotrophic fungal pathogens [22], and rice ONAC083 represses resistance to *Magnaporthe oryzae* by activating transcription of the RING-H2 gene OsRFPH2-6 [23]. Collectively, these studies highlight the diverse regulatory roles of NAC TFs in plant immunity across various plant–pathogen interaction systems.

Although the pepper NAC gene family has been systematically identified [24], the transcriptional dynamics and regulatory roles of NAC TFs during pepper–*P. capsici* interactions remain poorly elucidated [25,26]. In this study, we screened and identified three NAC transcription factors—CaNAC61, CaNAC79, and CaNAC92—exhibiting significant upregulation at distinct infection stages, based on our RNA-seq data from pepper during *P. capsici* infection. Using gain-of-function and loss-of-function strategies, we further demonstrated that CaNAC61, CaNAC79, and CaNAC92 negatively regulate pepper resistance to *P. capsici*. These findings provide valuable functional insights and potential candidate target genes for dissecting the immune regulatory network and advancing genetic improvement for Phytophthora blight resistance.

## 2. Materials and Methods

### 2.1. Plant Materials, Pathogen, and Culture Conditions

Pepper plants were cultivated in plastic pots filled with a substrate mixture of perlite and peat moss (1:2, *v*/*v*) in an artificial climate chamber under controlled conditions: 25 °C, 16 h light/8 h dark photoperiod, relative humidity of approximately 70%, and light intensity of 60–70 mmol photons m^−2^ s^−1^ [27]. *Phytophthora capsici* strain JX1 was initially cultured on 10% V8 agar medium in the dark at 25 °C. Subsequently, the mycelia were transferred to 10% V8 liquid medium and further incubated in the dark at 25 °C for 3 days. To induce zoospore release, the mycelia were intermittently washed three times with sterile water at 30 min intervals and then incubated at 25 °C until zoospores were released. The concentration of zoospores was adjusted to ~5 × 10^5^ zoospores·mL^−1^ before inoculation [28].

### 2.2. RNA-seq Data and Expression Profiling

RNA-seq data were retrieved from our prior study and are accessible in the NCBI Sequence Read Archive (SRA) under the accession number PRJNA627546 [27]. Briefly, the pepper line CM334 was hydroponically cultured with Hoagland nutrient solution until the five-true-leaf stage under the aforementioned growth conditions [27]. Roots were immersed in the zoospore suspension, and root samples were harvested at 0, 3, 6, 12, 24, 48, and 72 h post-inoculation (hpi). Three independent biological replicates were harvested at each time point for total RNA extraction and cDNA library construction. Paired-end libraries (PE150) were sequenced on an Illumina HiSeq2000 platform (Illumina, San Diego, CA, USA). Raw reads were quality-filtered using fastp (v0.23.2) and aligned to the pepper reference genome (CM334 v1.55) using HISAT2 (v2.2.1). Differential expression analysis was performed using DESeq2 (v1.36.0). Differentially expressed genes (DEGs) were identified using the criteria of |log_2_(fold change)| > 1 and FDR < 0.01.

### 2.3. RNA Extraction and RT-qPCR Analysis

Total RNA was extracted from plant tissues using TRIzol reagent (Invitrogen, Carlsbad, CA, USA) according to the manufacturer’s protocol. First-strand cDNA was synthesized with an All-in-One First-Strand cDNA Synthesis Super Mix kit (Yugong Biotech, Lianyungang, China). RT-qPCR assays were conducted using SYBR Green qPCR Master Mix with gene-specific primers (Table S1). Primer specificity was confirmed by melt-curve analysis, which yielded a single sharp peak for each primer pair, indicating no primer-dimer or non-specific amplification. Each treatment included three independent biological replicates. Relative transcript levels were computed using the 2^−ΔΔCt^ method [29] and normalized to the housekeeping gene *CaActin* [27].

### 2.4. Phylogenetic Relationships and Conserved Domain Analysis

To investigate the evolutionary relationships of CaNAC61, CaNAC79, and CaNAC92 within the NAC transcription factor family, homologous NAC protein sequences from *Arabidopsis thaliana*, *Solanum lycopersicum*, *Nicotiana benthamiana*, *Glycine max*, *Oryza sativa*, and *Zea mays* were retrieved from the NCBI database. These sequences, together with CaNAC61, CaNAC79, and CaNAC92, were used for phylogenetic analysis. Multiple sequence alignment was carried out using ClustalW 2.1 with default parameters. A phylogenetic tree was constructed using the maximum likelihood (ML) method in MEGA 12 software, with 1000 bootstrap replicates to evaluate the statistical confidence of each branch. To further assess the sequence conservation of CaNAC61, CaNAC79, and CaNAC92, multiple sequence alignment was also performed using DNAMAN 10 software, and the conserved NAC domains were identified and characterized in detail.

### 2.5. Subcellular Localization

For subcellular localization analysis, the open reading frames (ORFs) of *CaNAC61*, *CaNAC79*, and *CaNAC92* (without the stop codon) were individually cloned into the pBinGFP3 vector to generate C-terminal GFP fusion constructs. The resultant plasmids (35S:CaNAC61-GFP, 35S:CaNAC79-GFP, and 35S:CaNAC92-GFP), as well as the empty GFP vector (served as a negative control) and 35S:H2B-RFP (served as a nuclear marker), were transformed into Agrobacterium tumefaciens strain GV3101. The agrobacterial cultures harboring the GFP construct and the H2B-RFP construct were respectively resuspended in an induction buffer (10 mM MgCl_2_, 10 mM MES, 200 μM acetosyringone, pH 5.6) to an OD_600_ of 0.8, mixed thoroughly at a 1:1 (*v*/*v*) ratio and then infiltrated into *N. benthamiana* leaves via a needleless syringe. Fluorescence signals were visualized using a confocal laser scanning microscope (TCS SP8, Leica, Solms, Germany) at 48 h post-agroinfiltration (hpi) as described in our previous reports [30,31].

### 2.6. Transient Overexpression Assays in Pepper Leaves

Transient overexpression assays were conducted as previously described [31]. Agrobacterial cultures harboring the GFP fusion construct were resuspended in the induction buffer (10 mM MgCl_2_, 10 mM MES, 200 μM acetosyringone, pH 5.6) to an OD_600_ of 0.8 and then infiltrated into the third fully expanded leaves of eight-leaf-stage pepper plants. At 2 days post-infiltration (dpi), the infiltrated leaves were detached and inoculated with *P. capsici* zoospore suspension for disease assessment [30,31].

### 2.7. Virus-Induced Gene Silencing (VIGS) in Pepper

Virus-induced gene silencing was performed using the tobacco rattle virus (TRV)-based system as previously described [27]. To construct VIGS vectors, gene-specific silencing fragments of 314, 334, and 289 bp were designed for *CaNAC61*, *CaNAC79*, and *CaNAC92*, respectively, using the VIGS tool in the Sol Genomic Network (SGN) website (https://solgenomics.net). No off-target gene (which shares no more than a 19 bp matching fragment) was detected in the pepper genome cDNA database. The sequences of these silencing fragments are provided in Table S2. Then, these gene-specific fragments were individually cloned into the pTRV2 vector to generate recombinant constructs pTRV2: *CaNAC61*, pTRV2: *CaNAC79*, and pTRV2: *CaNAC92*. *A. tumefaciens* cultures harboring pTRV1, the respective pTRV2 derivatives (pTRV2: *CaNAC61/79/92*), as well as pTRV2:0 (empty vector, negative control) and pTRV2: *PDS* (indicator control), were resuspended in the induction buffer to an OD_600_ of 0.8. Equal volumes of TRV1 and each TRV2 culture (1:1, *v*/*v*) were mixed and incubated at 28 °C with gentle shaking (60 rpm) in the dark for 3 h. The mixed bacterial suspensions were infiltrated into cotyledons of 2–3 leaf-stage pepper seedlings. Following agroinfiltration, the seedlings were maintained at 16 °C in the dark for 56 h and then transferred to standard growth conditions. At approximately 3–4 weeks post-infiltration, the occurrence of photobleaching symptoms in TRV: *PDS*-infiltrated plants confirmed successful VIGS, and the silencing efficiencies of target *CaNAC* genes were further verified by RT-qPCR [30]. To verify the silencing specificity, RT-qPCR analyses were performed to assess the transcript levels of all three *CaNAC* genes in each VIGS treatment, which confirmed that silencing of any individual gene did not significantly affect the transcript levels of the other two genes (Figure S1).

### 2.8. Generation of Transgenic N. benthamiana Lines

To generate stable overexpression lines, the ORFs of *CaNAC61*, *CaNAC79*, and *CaNAC92* were individually cloned into the binary vector pK7WG2, which is driven by the constitutive CaMV 35S promoter. The resultant constructs were transformed into *A. tumefaciens* strain GV3101 and then transformed into *N. benthamiana* via an *Agrobacterium*-mediated leaf-disc transformation method [30]. Kanamycin-resistant regenerated plants were selected to obtain independent T_0_ transgenic plants, which were further confirmed by PCR amplification with transgene-specific primers. A total of 17, 18, and 23 independent T_0_ transgenic *N. benthamiana* lines were obtained for *CaNAC61*, *CaNAC79*, and *CaNAC92* transformants, respectively. All T_0_ lines were advanced to the T_1_ generation. Segregation analyses were performed to identify lines following the expected 3:1 Mendelian segregation ratio. Total RNA was isolated from T_1_ transgenic lines and reverse-transcribed into cDNA. RT-PCR was then performed to assess the expression levels of the target *CaNAC* genes, with *NbActin* used as the internal reference gene. For each *CaNAC* transformant, two independent T_1_ transgenic lines with high levels of transgene expression were selected for subsequent phenotyping assays [27].

### 2.9. P. capsici Infection Assays and Disease Evaluation

For the detached leaf inoculation assay, fully expanded leaves of pepper or *N. benthamiana* were placed on trays lined with pre-moistened tissue paper and inoculated with 5 μL of zoospore suspension (~5 × 10^5^ zoospores mL^−1^). The trays were sealed with plastic wrap to maintain high humidity and incubated in the dark at 25 °C for 2–3 days prior to disease evaluation [30]. Lesions on inoculated leaves were visualized under UV illumination and measured with a ruler. For the root inoculation assay, pepper or transgenic *N. benthamiana* plants were irrigated with 5 mL of the *P. capsici* zoospore suspension (~5 × 10^5^ zoospores mL^−1^). Disease indexes were recorded at the indicated days post-inoculation (dpi) as previously described [32]. Briefly, disease indexes were scored using a 0–5 scale: 0, asymptomatic; 1, basal stems slightly blackened with no leaf wilting; 2, basal stems blackened by 1–2 cm with some leaves wilted; 3, basal stems blackened by >2 cm with approximately half of the leaves wilted; 4, basal stems blackened and constricted with most leaves wilted; and 5, whole plant withered or dead. Each experiment was repeated twice, with six independent biological replicates per treatment.

### 2.10. Statistical Analysis

Statistical analyses were performed using SPSS 21.0, and data are presented as the mean ± standard deviation (SD). The data were analyzed by Student’s *t*-test, with statistical significance defined as *p* < 0.05 or *p* < 0.01.

## 3. Results

### 3.1. CaNAC61, CaNAC79, and CaNAC92 Are Induced by P. capsici Infection

**Figure 1:**
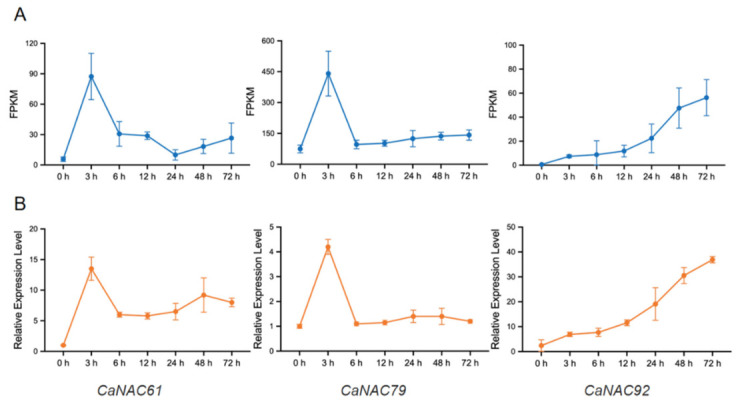
Expression patterns of *CaNAC61*, *CaNAC79*, and *CaNAC92* during *P. capsici* infection in pepper: (**A**) Transcriptional patterns of *CaNAC61*, *CaNAC79*, and *CaNAC92* at 0, 3, 6, 12, 24, 48, and 72 h post-inoculation with *P. capsici* were determined by RNA-seq. (**B**) The expression patterns of *CaNAC61*, *CaNAC79*, and *CaNAC92* at different time points post-inoculation were validated by RT-qPCR. Data are presented as the mean ± standard deviation (SD) from three independent biological replicates.

To identify NAC transcription factors involved in pepper’s defense response against *P. capsici* infection, we analyzed the transcriptional profiles of NAC family members using our previously published RNA-seq data from the pepper–*P. capsici* interaction [27]. We identified eight differentially expressed *CaNAC* genes in the RNA-seq dataset; among these, *CaNAC61*, *CaNAC79*, and *CaNAC92* were selected for further analysis due to their significant upregulation during infection (Figure 1A and Figure S2). Specifically, *CaNAC61* and *CaNAC79* displayed an early and transient induction pattern, with transcript levels rapidly peaking at 3 h post-inoculation (hpi) and subsequently declining to relatively low levels from 6 to 72 hpi. In contrast, *CaNAC92* exhibited a sustained late-phase induction pattern, with transcript levels gradually increasing during the infection and showing robust upregulation after 24 hpi, remaining at high levels at 48–72 hpi (Figure 1A). Thus, *CaNAC61* and *CaNAC79* represented early-responsive NAC genes, whereas *CaNAC92* represented a late-responsive NAC gene, suggesting that these CaNAC members may function at different stages of the pepper defense response.

To validate the RNA-seq data, RT-qPCR analysis was performed to examine the transcript profiles of *CaNAC61*, *CaNAC79*, and *CaNAC92* at different time points post-inoculation. The expression profiles revealed by RT-qPCR were largely consistent with the RNA-seq data, confirming the early and robust induction of *CaNAC61/79* and the sustained induction of *CaNAC92* during the infection (Figure 1B). These findings suggested that CaNAC61, CaNAC79, and CaNAC92 are pathogen-responsive transcription factors, implying that they may participate in the regulation of pepper defense response against *P. capsici* infection.

### 3.2. Phylogenetic Relationships and Conserved Domain Analysis of CaNAC61, CaNAC79, and CaNAC92

**Figure 2:**
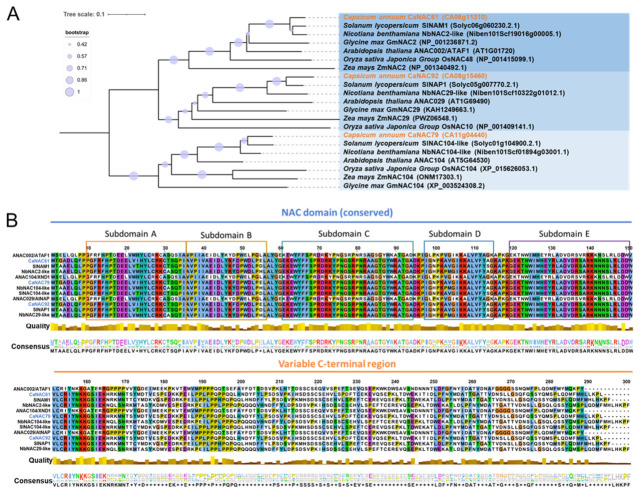
Phylogenetic relationships and conserved domain analysis of CaNAC61, CaNAC79, and CaNAC92: (**A**) A phylogenetic tree was constructed using NAC protein sequences from *Capsicum annuum*, *Solanum lycopersicum*, *Nicotiana benthamiana*, *Glycine max*, *Arabidopsis thaliana*, *Oryza sativa*, and *Zea mays*. CaNAC61, CaNAC79, and CaNAC92, which are highlighted in orange, were clustered into distinct phylogenetic clades with representative homologous NAC proteins from other plant species. Bootstrap values are represented by the size of the circles, and the scale bar indicates evolutionary distance. (**B**) Multiple sequence alignment was performed for CaNAC61, CaNAC79, and CaNAC92 with their homologous proteins. Sequence alignment revealed that all three proteins contain a conserved N-terminal NAC domain, a characteristic feature of the NAC transcription factor family that can be divided into five subdomains.

To investigate the evolutionary relationships of CaNAC61, CaNAC79, and CaNAC92 within the NAC transcription factor family, a phylogenetic tree was constructed using NAC protein sequences from a broader range of plant species, including *Solanum lycopersicum*, *Nicotiana benthamiana*, *Glycine max*, *Arabidopsis thaliana*, *Oryza sativa*, and *Zea mays*. The results showed that CaNAC61, CaNAC79, and CaNAC92 clustered into distinct clades together with their homologous NAC proteins from other plant species (Figure 2A).

To further examine the structural conservation of these NAC proteins, multiple sequence alignment was performed. The alignment revealed that CaNAC61, CaNAC79, and CaNAC92 harbor a conserved NAC domain at the N-terminus, which represents a canonical structural feature of NAC transcription factors [16]. The NAC domain could be further divided into five highly conserved subdomains (A–E) (Figure 2B). Among these subdomains, subdomains A, C, and D exhibited remarkable sequence conservation, whereas the C-terminal regions showed relatively high divergence.

### 3.3. CaNAC61, CaNAC79, and CaNAC92 Localize to the Nucleus

**Figure 3:**
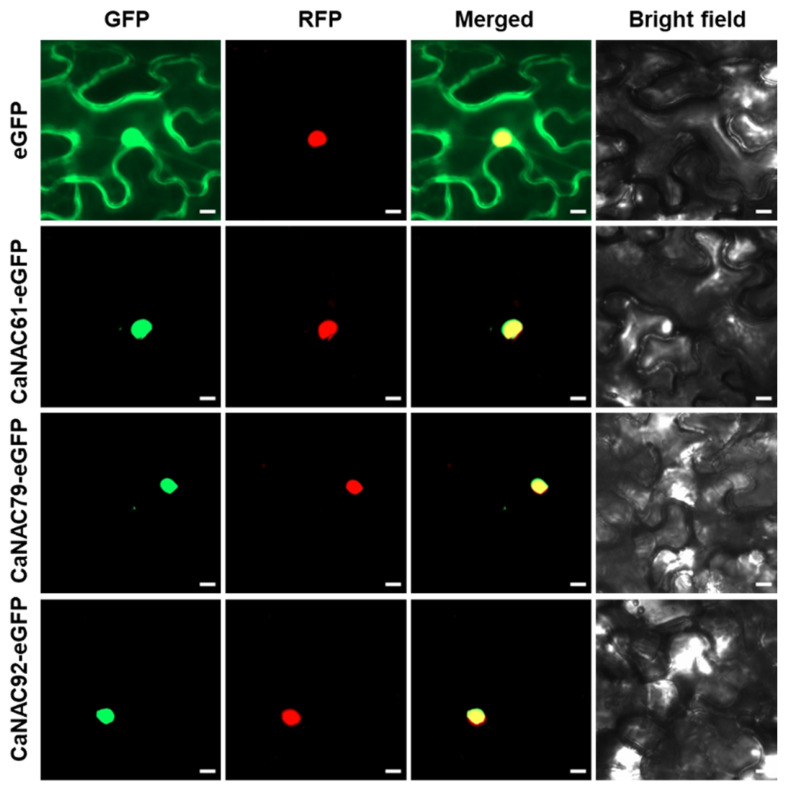
Subcellular localization of CaNAC61, CaNAC79, and CaNAC92. CaNAC61-eGFP, CaNAC79-eGFP, and CaNAC92-eGFP fusion proteins were transiently expressed in *N. benthamiana* leaves and co-expressed with the nuclear marker H2B-RFP. Confocal microscopy revealed that the GFP signals of CaNAC61, CaNAC79, and CaNAC92 strongly co-localized with the RFP signals, indicating that all three proteins are predominantly localized in the nucleus. eGFP was used as the empty vector control, showing fluorescence signals in both the nucleus and cytoplasm. GFP, green fluorescent protein. RFP, red fluorescent protein. Bar = 10 μm.

To determine the subcellular distribution of these NAC proteins, we generated GFP fusion constructs CaNAC61-GFP, CaNAC79-GFP, and CaNAC92-GFP driven by the constitutive CaMV 35S promoter and transiently expressed each of them in *N. benthamiana* leaves. Fluorescence microscopy observations revealed that the GFP signals of all three fusion proteins were exclusively localized in the nucleus, where they overlapped perfectly with the nuclear marker H2B-RFP. In contrast, the free GFP control showed fluorescence throughout the cytoplasm and nucleus (Figure 3).

### 3.4. Silencing of CaNAC61, CaNAC79, or CaNAC92 Enhances Pepper Resistance to P. capsici

**Figure 4:**
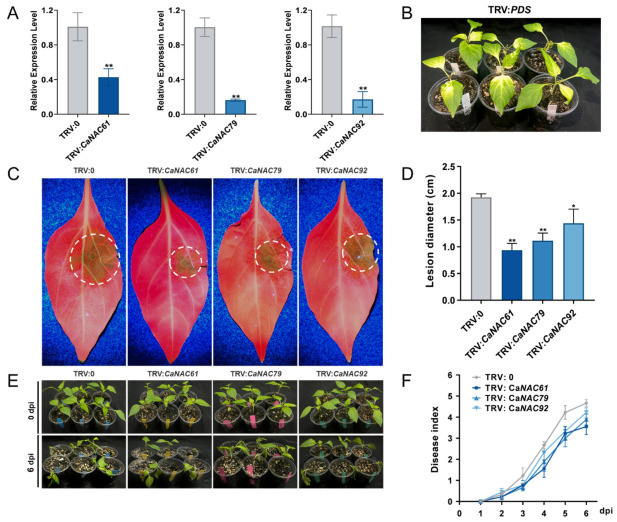
Silencing of *CaNAC61*, *CaNAC79*, and *CaNAC92* enhances resistance to *P. capsici* in pepper: (**A**) Transcript levels of *CaNAC61*, *CaNAC79*, and *CaNAC92* in TRV-mediated gene-silenced pepper plants were measured by RT-qPCR, with TRV: 0 serving as the negative control. Data represent the mean ± SD of three independent biological replicates. (**B**) Approximately 3–4 weeks post-agroinfiltration, TRV: *PDS*-treated plants exhibited typical photobleaching, confirming the effectiveness of the VIGS system. (**C**) Representative disease symptoms of detached VIGS leaves at 3 days post-inoculation (dpi) with *P. capsici* zoospores. (**D**) Lesion diameters after *P. capsici* inoculation at 3 dpi. (**E**) Representative disease symptoms of TRV-treated pepper plants subjected to root inoculation at 0 dpi and 6 dpi. (**F**) Disease indexes of these TRV-treated pepper plants after root inoculation with *P. capsici* zoospores. Data are presented as the mean ± SD from six independent biological replicates. Asterisks indicate significant differences based on Student’s *t*-test (* *p* < 0.05, ** *p* < 0.01).

To further dissect the roles of *CaNAC61*, *CaNAC79*, and *CaNAC92* in pepper immunity against *P. capsici*, a tobacco rattle virus (TRV)-based virus-induced gene silencing (VIGS) system was employed to specifically silence each of these three *CaNAC* genes in pepper plants. Approximately 3~4 weeks post-agroinfiltration, the TRV: *PDS*-treated plants exhibited typical photobleaching, and RT-qPCR analysis confirmed that the transcript levels of *CaNAC61*, *CaNAC79*, and *CaNAC92* were effectively down-regulated in the corresponding TRV: *CaNAC61*, TRV: *CaNAC79*, and TRV: *CaNAC92* plants, as compared with the TRV: 0 control plants (Figure 4A,B).

Detached leaves from the silenced pepper plants were subsequently challenged with *P. capsici*, and the inoculation assays revealed that lesion diameters were significantly reduced in the *CaNAC61*-, *CaNAC79*-, and *CaNAC92*-silenced leaves relative to the TRV: 0 control leaves (Figure 4C,D). Similarly, in the root-inoculation assays, *CaNAC61*-, *CaNAC79*-, and *CaNAC92*-silenced pepper plants exhibited milder disease symptoms and lower disease indexes compared with TRV: 0 control plants (Figure 4E,F). These results suggest that CaNAC61, CaNAC79, and CaNAC92 function as negative regulators of pepper resistance against *P. capsici*.

### 3.5. Transient Overexpression of CaNAC61/79/92 in Pepper Leaves Enhances Susceptibility to P. capsici

**Figure 5:**
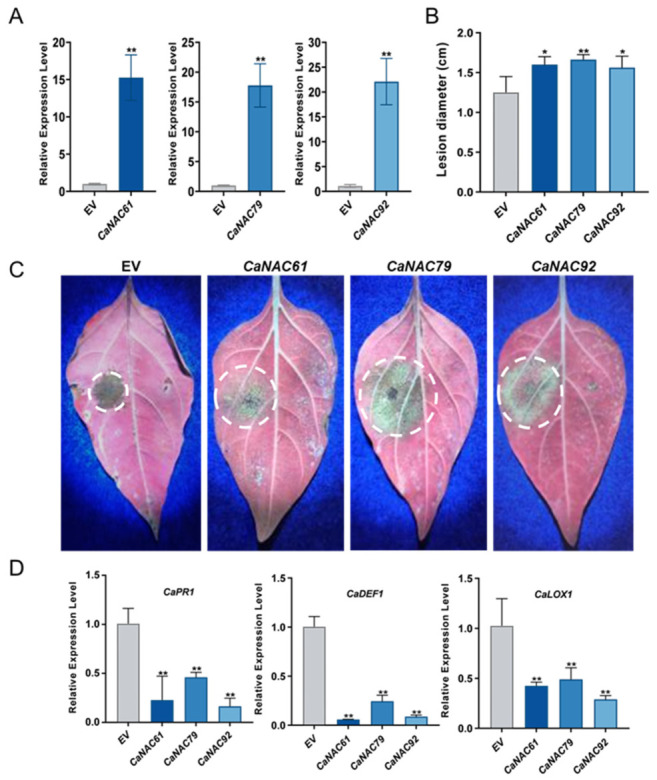
Transient overexpression of *CaNAC61*, *CaNAC79*, and *CaNAC92* enhances susceptibility of pepper leaves to *P. capsici* infection: (**A**) Transcript levels of *CaNAC61*, *CaNAC79*, and *CaNAC92* in transiently overexpressing pepper leaves were measured by RT-qPCR. The empty vector (EV) served as the control. Data represent the mean ± SD of three independent biological replicates. (**B**) Lesion diameters in pepper leaves at 2 days post-inoculation (dpi) with *P. capsici* zoospores. Data are presented as the mean ± SD from six independent biological replicates. (**C**) Representative disease symptoms of pepper leaves following *P. capsici* inoculation at 2 dpi. (**D**) Expression of defense marker genes in pepper leaves transiently overexpressing *CaNAC61*, *CaNAC79*, or *CaNAC92*. Transcript levels of *CaPR1*, *CaDEF1*, and *CaLOX1* were determined by RT-qPCR at 2 days post-agroinfiltration. Data were obtained from three independent biological replicates. The empty vector (EV) served as the control. All data are presented as the mean ± SD, and asterisks indicate significant differences based on Student’s *t*-test (* *p* < 0.05, ** *p* < 0.01).

Given that stable genetic transformation in pepper is highly challenging, we employed an *Agrobacterium*-mediated transient expression system to individually overexpress *CaNAC61*, *CaNAC79*, and *CaNAC92* in pepper leaves and investigate their roles during *P. capsici* infection. At 2 days post-agroinfiltration, successful overexpression of *CaNAC61*, *CaNAC79*, and *CaNAC92* in the infiltrated leaves was confirmed by RT-qPCR (Figure 5A). The overexpressing leaves were then challenged with *P. capsici* zoospores to assess disease symptoms. Compared with the empty-vector (EV) control, pepper leaves overexpressing *CaNAC61*, *CaNAC79*, or *CaNAC92* developed significantly larger lesions upon *P. capsici* inoculation (Figure 5B,C), suggesting that overexpression of these NAC genes enhances pepper susceptibility to the pathogen.

To test whether transient overexpression of *CaNAC61*, *CaNAC79*, or *CaNAC92* alters defense-related gene expression in pepper plants, we examined the transcript levels of defense-related marker genes, including *CaPR1*, *CaPR2*, *CaPR10*, *CaDEF1*, *CaLOX1*, and *CaACO1* [31,33]. Compared with the empty-vector control, the transcript levels of *CaPR1*, *CaDEF1*, and *CaLOX1* were significantly downregulated in leaves overexpressing any of the three *CaNAC* genes (Figure 5D). However, *CaPR2* was specifically repressed only by *CaNAC61* overexpression, *CaPR10* was uniquely downregulated by *CaNAC92* overexpression, and *CaACO1* expression remained largely unchanged across all three overexpression assays (Figure S3).

### 3.6. Stable Overexpression of CaNAC61/79/92 in N. benthamiana Increases Susceptibility to Phytophthora Blight

**Figure 6:**
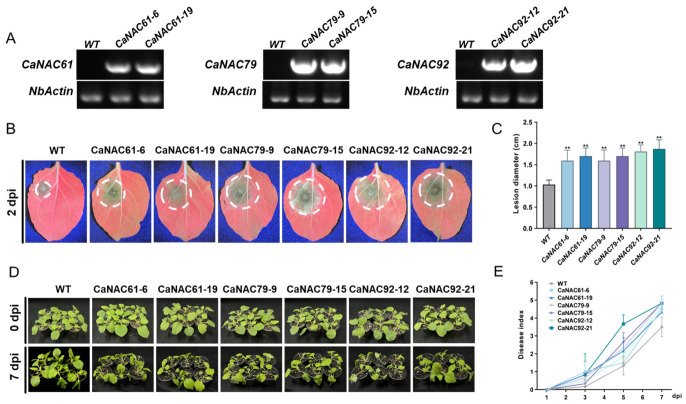
Ectopic stable overexpression of *CaNAC61*, *CaNAC79*, and *CaNAC92* enhances susceptibility to *P. capsici* in *N. benthamiana*: (**A**) RT-PCR analysis of *CaNAC61*, *CaNAC79*, and *CaNAC92* transcript levels in transgenic *N. benthamiana* lines overexpressing each of these genes. *NbActin* was used as the internal reference, and wild-type (WT) plants served as the control. (Figure S4). (**B**) Representative disease symptoms of detached leaves from different transgenic *N. benthamiana* lines following *P. capsici* inoculation at 2 dpi. (**C**) Lesion diameters of these transgenic *N. benthamiana* leaves after *P. capsici* inoculation at 2 dpi. (**D**) Representative disease symptoms of these transgenic *N. benthamiana* plants subjected to root inoculation at 0 dpi and 7 dpi. (**E**) Disease indexes of different transgenic *N. benthamiana* lines after root inoculation with *P. capsici* zoospores. The data are presented as the mean ± SD from six independent biological replicates. Asterisks indicate significant differences based on Student’s *t*-test (** *p* < 0.01).

Because pepper is very recalcitrant to stable genetic transformation, we generated transgenic *N. benthamiana* lines overexpressing *CaNAC61*, *CaNAC79*, or *CaNAC92* to further validate their functional roles against *P. capsici* infection. RT-PCR analysis confirmed successful overexpression of the three NAC genes in the corresponding transgenic lines (Figure 6A). Under normal growth conditions, transgenic lines overexpressing *CaNAC61*, *CaNAC79*, or *CaNAC92* showed no obvious differences in growth or morphology compared with wild-type (WT) plants (Figure 6D). In detached leaf inoculation assays, leaves from these transgenic lines developed significantly larger lesions than those from WT plants following *P. capsici* inoculation (Figure 6B,C). In root inoculation assays, WT plants developed severe disease symptoms following *P. capsici* infection. In contrast, transgenic lines overexpressing *CaNAC61*, *CaNAC79*, or *CaNAC92* exhibited markedly enhanced susceptibility, as evidenced by more severe disease symptoms and higher disease indexes (Figure 6D,E). All these data demonstrated that ectopic overexpression of *CaNAC61*, *CaNAC79*, or *CaNAC92* compromises resistance to *P. capsici*, further validating their roles as negative regulators in defense response against the pathogen.

## 4. Discussion

Phytophthora blight, caused by *P. capsici*, is one of the most devastating diseases threatening pepper production worldwide [3,5,6]. This pathogen exhibits a broad host range and spreads rapidly under conducive environmental conditions, often causing severe plant wilting and mortality, leading to substantial losses in both yield and fruit quality [3]. Moreover, germplasm resources for resistance to *P. capsici* in pepper are inadequate, which greatly hinders the breeding of stable and durable resistant cultivars [4]. Thus, screening and identifying key regulators in pepper defense response against *P. capsici* is critical for elucidating plant–pathogen interactions and accelerating the breeding of disease-resistant varieties.

NAC transcription factors comprise a large family of plant-specific transcriptional regulators characterized by a conserved NAC domain at the N-terminus and a variable transcriptional regulatory region at the C-terminus [14]. Accumulating evidence has demonstrated that NAC transcription factors participate not only in plant development and abiotic stress responses but also in the regulation of plant immunity [12,17]. Although the NAC gene family in pepper has been identified in genome-wide analyses, and the expression patterns of several NAC genes in response to *P. capsici* infection have been documented [24], the functional role of NAC transcription factors in pepper resistance against *P. capsici* remains largely unclear.

In this study, our findings indicated that CaNAC61, CaNAC79, and CaNAC92 act as negative regulators of pepper immunity against *P. capsici*. Similar negative regulatory roles have been reported for NAC transcription factors in diverse plant–pathogen interaction systems. For example, in rice, the NAC transcription factor ONAC083 negatively regulates resistance to *Magnaporthe oryzae* by directly activating the expression of the RING-H2-type E3 ubiquitin ligase gene *OsRFPH2-6* [23]. In wheat, TaNAC1 has also been shown to reduce resistance to stripe rust (*Puccinia striiformis* f. sp. *tritici*) and to suppress the expression of defense-related genes such as PR1, PR2, and WRKY70, indicating a similar negative regulatory role in plant immunity [34]. Moreover, in *Arabidopsis*, a regulatory module composed of *NAC90*, *NAC36*, and their interacting partner *NAC61* restricts systemic immune responses by repressing N-hydroxypipecolic acid (NHP) biosynthesis [35]. Notably, a pepper NAC member, CaNAC4, has recently been implicated in the negative regulation of biotic stress responses, as *CaNAC4*-overexpressing transgenic *N. benthamiana* plants displayed enhanced susceptibility to *Botrytis cinerea* and *Pseudomonas syringae* pv. *tabaci* [36]. Together with these previous findings, our results extend the negative regulatory roles of NAC transcription factors in the pepper–*P. capsici* pathosystem.

Given that the crosstalk between NAC transcription factors and hormone signaling networks has been reported in multiple plant species [17,24], CaNAC61, CaNAC79, and CaNAC92 may also influence immune output through the regulation of defense-related hormone signaling pathways, such as SA, JA, and ET. In this study, we found that overexpression of *CaNAC61*, *CaNAC79*, or *CaNAC92* significantly suppresses the transcript levels of the SA-responsive gene *CaPR1* and the JA-responsive genes *CaDEF1* and *CaLOX1* but cannot significantly affect the ET-associated gene *CaACO1* (Figure 5D and Figure S3), indicating that these three TFs likely attenuate plant immunity through SA- and JA-mediated defense pathways. Further investigation is needed to identify the direct targets of these CaNAC TFs and to elucidate the fundamental mechanisms underlying their negative regulation of pepper immunity against *P. capsici*.

As reported by Diao et al. [24], CaNAC61, CaNAC79, and CaNAC92 belong to different phylogenetic subgroups of the pepper CaNAC family. CaNAC61 was classified into the Group I ATAF subfamily. Notably, certain members of this subgroup—such as *Arabidopsis* ATAF1—have been shown to act as negative regulators of plant defense responses against both necrotrophic fungal and bacterial pathogens [22]. CaNAC92 was assigned to the Group I (4) subgroup, which exhibits high similarity to the AtNAP subgroup [24]. In contrast, CaNAC79 was classified into Group II, which exhibits relatively divergent sequence and motif features compared with Group I [24]. Moreover, *CaNAC61*, *CaNAC79*, and *CaNAC92* displayed distinct expression dynamics during the *P. capsici* infection. Specifically, *CaNAC61*/*79* were identified as early-responsive genes, whereas *CaNAC92* was a late-responsive gene (Figure 1). Although the transcript levels of *CaPR1*, *CaDEF1*, and *CaLOX1* were significantly downregulated by all three *CaNAC* genes (Figure 5D), the other two SA-responsive genes—*CaPR2* and *CaPR10*—were specifically repressed by *CaNAC61* and *CaNAC92*, respectively (Figure S3). These results indicated that these three genes may not act in a fully redundant manner.

It is noteworthy that our previous work demonstrated that the WRKY transcription factors CaWRKY01-10 and CaWRKY08-4 act as positive regulators of pepper resistance against *P. capsici* [30]. Combined with the findings presented in this study, it is suggested that both positive and negative regulatory modules coexist within the transcriptional regulatory network governing pepper immune responses to *P. capsici*. While positive regulators rapidly activate defense responses, negative regulators may function to prevent excessive immune activation that could otherwise impair plant growth and metabolism. The coordinated interplay of these regulatory modules illustrates that pepper immunity against *P. capsici* is not a simple on–off process but instead a precisely tuned dynamic balance controlled by multilayered and sophisticated transcriptional regulatory networks [10]. Given the considerable potential of negative regulators and susceptibility-related genes in resistance breeding, precisely downregulating their expression or implementing targeted gene editing offers a promising strategy to enhance plant resistance against pathogens [37,38].

## 5. Conclusions

Through time-series transcriptome and RT-qPCR analysis of pepper plants challenged with *P. capsici*, we identified three NAC TF genes—*CaNAC61*, *CaNAC79*, and *CaNAC92*—that were upregulated at the infection stages. Among them, *CaNAC61* and *CaNAC79* displayed an early and transient induction pattern, with transcript levels rapidly peaking at 3 h post-inoculation (hpi). In contrast, *CaNAC92* exhibited a sustained late-phase induction pattern, with transcript levels gradually increasing during the infection and showing robust upregulation after 24 hpi, remaining at high levels at 48–72 hpi. Gain- and loss-of-function analyses further demonstrated that transient overexpression of *CaNAC61*, *CaNAC79*, and *CaNAC92* in pepper leaves significantly promotes lesion expansion, whereas silencing each of these genes in pepper confers enhanced resistance to *P. capsici*. Consistently, heterologous overexpression in transgenic *N. benthamiana* further validated *CaNAC61*, *CaNAC79*, and *CaNAC92* as negative regulators in resistance to *P. capsici*. Collectively, our findings indicated that *CaNAC61*, *CaNAC79*, and *CaNAC92* negatively regulate plant resistance to *P. capsici*, thereby broadening our knowledge of NAC transcription factor functions in plant immunity and offering novel insights into the sophisticated regulatory network governing pepper resistance to *P. capsici*.

## Data Availability

The data presented in this study are available in the article. Further information is available upon request from the corresponding author.

## Supplementary Materials

The following supporting information can be downloaded at: https://www.mdpi.com/article/10.3390/biology15120943/s1, Table S1: Primers used in this study; Table S2: Sequences of the silencing fragments; Figure S1: Specificity verification of VIGS-mediated silencing of *CaNAC61*, *CaNAC79*, and *CaNAC92*; Figure S2: RNA-seq analysis of *CaNAC* gene expression levels in pepper leaves after *P*. *capsici* infection; Figure S3: Expression analysis of additional defense-related marker genes in pepper leaves transiently overexpressing *CaNAC61*, *CaNAC79*, or *CaNAC92*; Figure S4: Original image for PCR.
