# Supplementary material for: CaNAC61, CaNAC79, and CaNAC92 Act as Negative Regulators in Pepper Defense Response Against Phytophthora capsici
Source: Biology (Basel). 2026 Jun 17;15(12):943. doi: 10.3390/biology15120943 (PMC13296315; doi:10.3390/biology15120943)
Supplement: Supplementary file 1 [file biology-15-00943-s001.zip › biology-4330290-supplementary.pdf]

Table S1. Primers used in this study.

Gene	Forward primers (5'→3')	Reverse primers (5'→3')
Primers used for construction of overexpression vector		
PBinGFP3-	ACGATAGCCGGTACCCCCGGGATGACAGGA	GCCCTTGCTCACCATCCCGGGAAGAGGCT
CaNAC61	GCAGATTTGCAATT	TGAGAAGGTGCATG
PBinGFP3-	ACGATAGCCGGTACCCCCGGGATGGGGGAT	GCCCTTGCTCACCATCCCGGGGTGCGGCA
CaNAC79	AGTAATGTCAACCTG	AACTAATTTCATCA
PBinGFP3-	ACGATAGCCGGTACCCCCGGGATGGTTGGA	GCCCTTGCTCACCATCCCGGGCTGGAATTG
CaNAC92	AAAATTAGCTCTGATC	AAATGCTGGATTCA
Primers used for construction of VIGS vector		
TRV2:	GGGGACAAGTTTGTACAAAAAAGCAGGCTT	GGGGACCACTTTGTACAAGAAAGCTGGGT
CaNAC61	CCTCTACCACCACAACCACCA	CAGAGGCTTGAGAAGGTGCAT
TRV2:	GGGGACAAGTTTGTACAAAAAAGCAGGCTT	GGGGACCACTTTGTACAAGAAAGCTGGGT
CaNAC79	CATAGCCGGAGGACACAAAGT	CAGCATGATAGCTCTGTGCCA
TRV2:	GGGGACAAGTTTGTACAAAAAAGCAGGCTT	GGGGACCACTTTGTACAAGAAAGCTGGGT
CaNAC92	CATGAGGTTAGATGATTGGGT	CGTTCGTTGTGTAAATTTGGT
Primers for RT-qPCR analysis		
CaNAC61	TTGCAATTCCCTCCAGGCTT	GATTTGGGCGTGAGCCATTC
CaNAC79	TCCATCGCAAGGTTGCTCTT	GTTGCCTTCTGCCATTGCTT
CaNAC92	AGCTCTGATCTTCCTCCTGGAT	GCATGGCCTTGATGTAGCTTG
CaPR1	GCCGTGAAGATGTGGGTCAATGA	TGAGTTACGCCAGACTACCTGAGTA
CaDEF1	GTGAGGAAGAAGTTTGAAAGAAAGTAC	TGCACAGCACTATCATTGCATACAATTC
CaLOX1	TCACTCCTCAACTGCAGACA	TCTCATCGCCATTCATCTGT
CaPR2	ATTGTTGTGTCCGAGAGTGG	TAGGCCTTCTTGGACTTCCC
CaPR10	TGGCAAATTTGAAGCTTCTGC	TGTGCACATTGTGTTCTTCCT
CaACO1	CCATTGTGGTCAACCTTGGC	GCATCGCTTCCTGGATTGTAA
CaActin	AGGGATGGGTCAAAAGGATGC	GAGACAACACCGCCTGAATAGC
NbActin	CAGAAAGGACCTCTACGGTAACAT	TCTGTGGACGATGGACGGAC
Primers used for transgenic vector construction		
pK7WG2-	GGGGACAAGTTTGTACAAAAAAGCAGGCTT	GGGGACCACTTTGTACAAGAAAGCTGGGT
CaNAC61	CATGACAGGAGCAGATTTGCAATT	CTTAAAGAGGCTTGAGAAGGTGCATG
pK7WG2-	GGGGACAAGTTTGTACAAAAAAGCAGGCTT	GGGGACCACTTTGTACAAGAAAGCTGGGT
CaNAC79	CATGGGGGATAGTAATGTCAACCTG	CCTAGTGCGGCAAACTAATTTCATCA
pK7WG2-	GGGGACAAGTTTGTACAAAAAAGCAGGCTT	GGGGACCACTTTGTACAAGAAAGCTGGGT
CaNAC92	CATGGTTGGAAAAATTAGCTCTGATC	CTCACTGGAATTGAAATGCTGGATTCA

Table S2. Sequences of the silencing fragments.

Gene	Fragment length (bp)	Silencing fragment sequence (5’-3’)
CaNAC61	314	CAGGCTTGCTGATGTTGATCGATCTGCTCGTAAGAACAACAATAGC<br>TTAAGGTTGGATGATTGGGTTTTATGCCGAATATACAACAAAAAAG<br>GATCGATTGAAAAGCATCGGAAAATGAACACCAGCTATATGGATAC<br>AGTTGAATCACCGGAGGATAAAAAACCAGAGATTCTACCCCC<br>TCTACCACCACAACCACCACAGCAACTGCACAACGATTACTTCTAC<br>CTGCCTTCAGATTCAGTTCCAAAGATCCACTCTGATTCAAGCTGTT<br>CGGAGCACGTACTTTCACCAGATTTTAC
CaNAC79	334	ATAGCCGGAGGACACAAAGTAGAATCACTGAAAACGGATACTGGA<br>AATCATTAGGAGTTGATGAACCAATATTCTCAAGTTCAAGTGACAG<br>TAATATTGGCATGAAGAAATACTATGCTTTTTACCTTGGTGAGAAAC<br>CTGAAGGTGCCAGAACCAATTGGGTTATGCAAGAATTTAGTCTTTC<br>TGACTCTTCTTCTGCTTCTGCAAGTAGCCGTTCATCTTCTAGGAGA<br>AAAAGCCGTTCCAAAATCGACTATAGCGGTTGGGTAATATGTCGTG<br>TATACGAGCGCAATTGTGATAATGATGACGACGATAATGGCACAGA<br>GCTATCATGCT
CaNAC92	289	ATGAGGTTAGATGATTGGGTACTTTGTAGAATTTATAAGAAGAAGA<br>ATTTGGGAAAAACTATGGAAATGATGAAAGTTGAAGGAGAAGAAG<br>ATGTAGTAGAAAGTCATCATCAAATTGTGAGTACTACAAATAATTCA<br>ATTGAAGTTATTGGTGGACCACAAACAATGAAATTACCAAGAATTT<br>GTTCATTGTCACATCTATTGGAGTTGGATTATTTTGGATCAATTCCAC<br>AATTACTAGGTGATAATAATTCATATGATCATGATGACCAAATTTACA<br>CAACGAAC

Figure S1

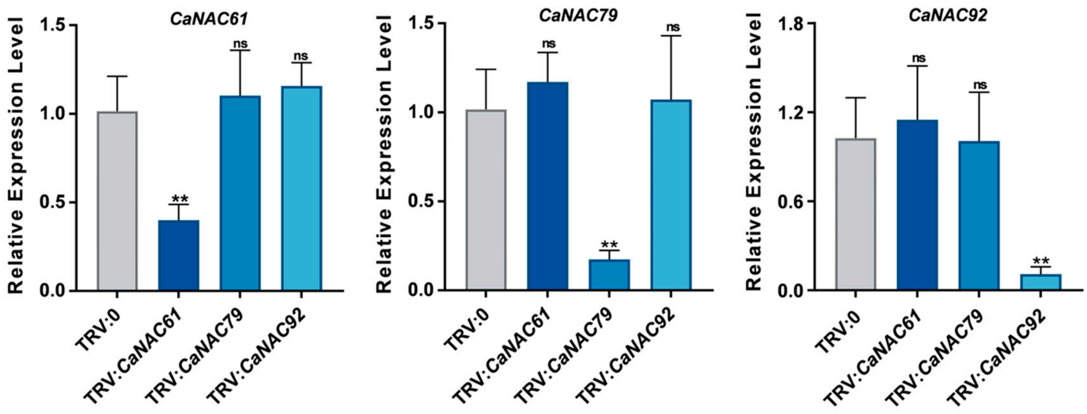

Figure S1. Specificity verification of VIGS-mediated silencing of *CaNAC61*, *CaNAC79*, and *CaNAC92*. The silencing efficiency was assessed by RT-qPCR analysis in TRV-mediated gene-silenced pepper plants. Data represent the mean ± SD from three independent biological replicates. Asterisks indicate significant differences based on Student's *t*-test (** $P < 0.01$; ns, not significant).

Figure S2

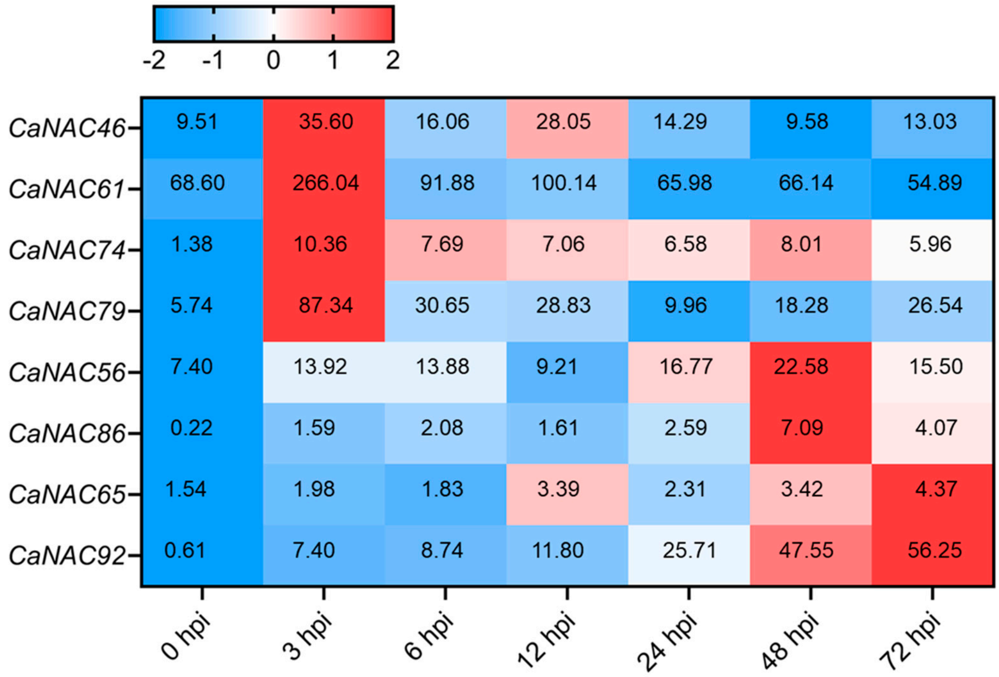

Figure S2. RNA-seq analysis of *CaNAC* gene expression levels in pepper leaves after *P. capsici* infection. The heatmaps of eight differentially expressed *CaNAC* genes (*CaNAC46*, *CaNAC61*, *CaNAC74*, *CaNAC79*, *CaNAC56*, *CaNAC86*, *CaNAC65*, and *CaNAC92*) at 0, 3, 6, 12, 24, 48, and 72 hours post inoculation (hpi). Expression values are presented as FPKM (fragments per kilobase of transcript per million mapped reads).

Figure S3

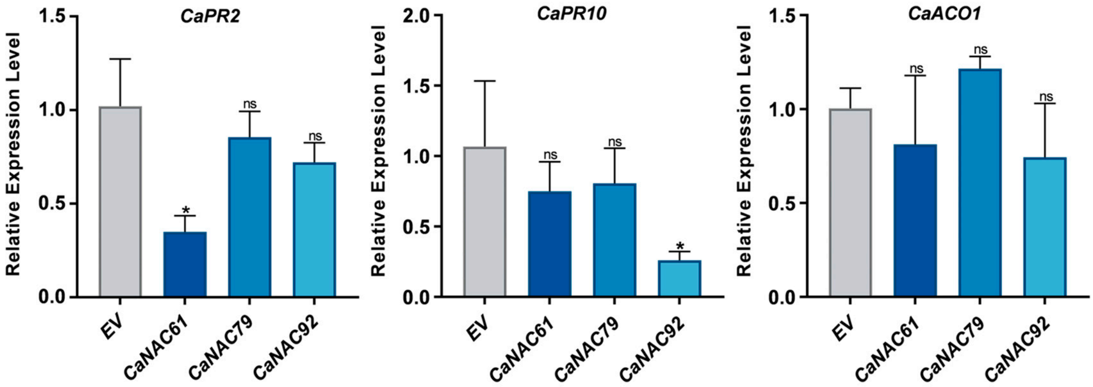

Figure S3. Expression analysis of additional defense-related marker genes in pepper leaves transiently overexpressing *CaNAC61*, *CaNAC79*, or *CaNAC92*. Transcript levels of *CaPR2*, *CaPR10*, and *CaACO1* were determined by RT-qPCR at 2 days post agroinfiltration. The empty vector (EV) served as the control. Data represent the mean ± SD of three independent biological replicates. Asterisks indicate significant differences based on Student's *t*-test (* $P < 0.05$; ns, not significant).
