# Supplementary figures and images for: CaNAC61, CaNAC79, and CaNAC92 Act as Negative Regulators in Pepper Defense Response Against Phytophthora capsici
Source: Biology (Basel). 2026 Jun 17;15(12):943. doi: 10.3390/biology15120943 (PMC13296315; doi:10.3390/biology15120943)

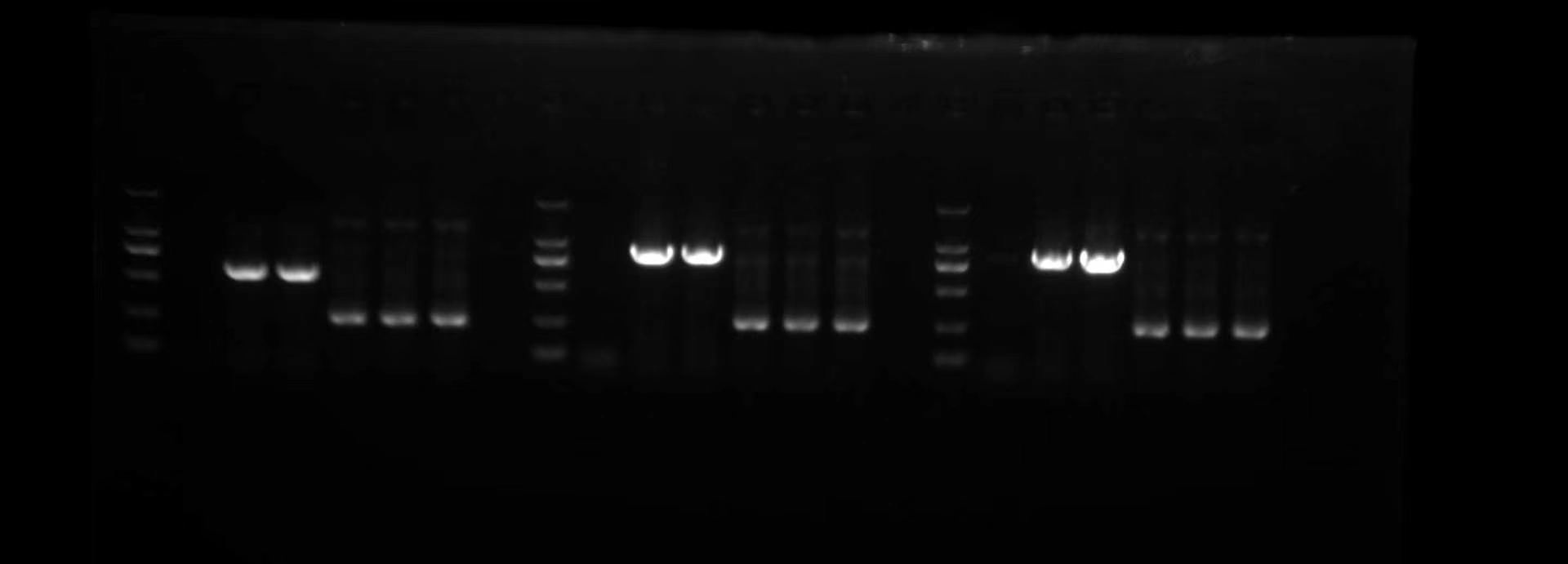

Supplement: Supplementary file 1 [file biology-15-00943-s001.zip › Figure S4.jpg]
